# Lycopene-Loaded Microemulsion Regulates Neurogenesis in Rats with A*β*-Induced Alzheimer's Disease Rats Based on the Wnt/*β*-catenin Pathway

**DOI:** 10.1155/2021/5519330

**Published:** 2021-09-06

**Authors:** Wen-jing Ning, Ren-jun Lv, Ning Xu, Xun-yao Hou, Chao Shen, Yun-liang Guo, Zhong-yu Fan, Na Cao, Xue-Ping Liu

**Affiliations:** ^1^Department of Geriatric Neurology, Shandong Provincial Hospital, Cheeloo College of Medicine, Shandong University, Jinan, Shandong 250012, China; ^2^First People's Hospital of Jinan, Jinan, Shandong 250000, China; ^3^Provincial Hospital Affiliated to Shandong First Medical University, Jinan, Shandong 250000, China; ^4^Department of Respiratory Medicine, The First School of Clinical Medicine, Lanzhou University, Lanzhou 730000, China; ^5^Shandong First Medical University&Shandong Academy of Medical Sciences, Jinan, Shandong 250000, China; ^6^Jigang Hospital of Jinan, Jinan, Shandong 250000, China

## Abstract

**Objective:**

To investigate the effects of lycopene-loaded microemulsion (LME) on the cognitive function and neurogenesis in the dentate gyrus (DG) of the hippocampus and subventricular (SVZ) region of rats with amyloid *β*- (A*β*-) induced Alzheimer's disease (AD) and its mechanism based on the Wnt/*β*-catenin pathway.

**Methods:**

Healthy Wistar rats were divided into four groups: the blank control (CON), AD control, traditional lycopene (LOO), and LME groups. The CON and AD groups were fed with normal saline, while the LOO group was fed with traditional lycopene, and the LME group was fed with lycopene-loaded microemulsion. Behavioral tests were performed after three weeks of gastric administration. Immunofluorescence-labeled cells were used to observe the differentiation and maturation of new nerve cells in the DG of the hippocampus and SVZ region. qRT-PCR and Western blotting detected the expression of neurogenesis genes and Wnt/*β*-catenin pathway-related proteins, respectively.

**Results:**

On the Morris water maze test, LME rats had significantly shortened movement trajectory on the searching platform, reduced escape latency time, and increased residence time on the original platform quadrant. In addition, more LME rats crossed the platform when it was removed. Thus, LME can improve the spatial learning and memory of A*β*-induced AD rats. On qRT-PCR, LME significantly increased Reelin, Nestin, and Pax6 gene expressions, which regulate neurogenesis. Immunofluorescence showed that LME could significantly increase BrdU^+^, Dcx^+^, BrdU^+^/Neun^+^, BrdU^+^/Dcx^+^ cells in the DG and SVZ regions, thus promoting neurogenesis. LME also reduced the number of Iba1^+^ and Iba1^+^/BrdU^+^ cells, thus reducing the neuroinflammatory response. On Western blot, LME upregulated the Wnt/*β*-catenin pathway by upregulating Wnt3a, *β*-catenin, Disheveled (Dvl), and p-GSK3*β* and downregulating p-*β*-catenin and GSK3*β*.

**Conclusion:**

LME attenuates cognitive impairment in A*β*-induced AD rats by promoting neurogenesis in the hippocampus and SVZ region through upregulating the Wnt/*β*-catenin pathway.

## 1. Introduction

Alzheimer's disease (AD) is a progressive degenerative disease of the central nervous system. It is one of the most common neurological diseases and often occurs in the elderly. The main clinical manifestations are progressive dementia, memory, and cognitive decline. The main pathological features are intracellular and extracellular amyloid *β* (A*β*) deposition and formation of intracellular neuronal tangles, which trigger neurodegeneration and neuronal loss [1]. Adult mammalian brain neurogenesis occurs at the subventricular zone(SVZ) and the lower levels of the hippocampal dentate gyrus (DG). Under certain conditions, two areas with the neural stem cells (NSCs) have the ability of differentiation, proliferation, and migration. Since these new neurons can develop in multiple directions, these can replace lost nerve cells in situ. Thus, making full use of endogenous NSCs can provide new means for AD treatment [2]. However, autologous NSCs have a limited ability to proliferate in situ under spontaneous conditions, and the mechanism of the brain's responses to A*β* injury remains unclear. Thus, neuroscientists face the issue of determining how to activate the proliferation of endogenous NSCs to complete the repair process. The Wnt/*β*-catenin signaling pathway gets its name because of its initial Wnt protein. It is widely distributed in various tissues and has functions in embryonic neural development, such as nerve cell proliferation, differentiation, fate determination, apoptosis, axon guidance, and synaptic formation [3–5]. The Wnt pathway plays an important role in the development of the embryonic nervous system [5]. Lycopene, which is found in tomatoes and other red fruits, belongs to the carotenoid family. As an effective antioxidant and singlet oxygen quenching agent, lycopene has many physiologic roles such as antioxidative stress, anti-inflammatory, antiapoptosis, and antitumor effects and promoting neurogenesis and protecting the nervous system. Microemulsion is a stable thermodynamic system formed by mixing oil phase, surfactant, cosurfactant, and water with particle size < 100 *μ*m. Studies have shown that microemulsions with a specific composition can improve the oral bioavailability of insoluble drugs and promote targeted brain transport, potentially improving neuroprotection and promoting neurogenesis [6, 7]. Lycopene-loaded microemulsion (LME) is prepared by the synthesis of lycopene and a nanoemulsion. Previous studies have shown that LME can significantly improve lycopene's oral absorption and bioavailability, reduce its elimination rate, and increase its retention time in blood circulation; it also has good brain targeting [6]. In this study, an A*β*-induced AD cognitive impairment model was established. Rats in each group were fed differently to observe behavior changes such as spatial learning ability and neurogenesis in the hippocampal DG and SVZ regions. The expression of the Wnt/*β*-catenin pathway-related proteins in hippocampal regions were also studied to explore the possible mechanism of LME on A*β*-induced AD model rats.

## 2. Materials and Methods

### 2.1. Reagents and Instruments

The main reagents and instruments included A*β*1-42 (Sigma, A9810, GER), lycopene (Shanxi Comsenz Biological Technology Co., Ltd.), mouse monoclonal antibody of BrdU (Sigma B5002, GER), rabbit monoclonal antibody of Dcx (Abcam, ab207175, UK), rabbit monoclonal antibodies Neun resistance (Abcam, ab177487, UK), rabbit monoclonal antibody of Iba1 (Abcam, ab178846, UK), *β*-actin antibody (Proteintech, PTG 20536-1-AP, China), Anti-Wnt3a antibody (Abcam, EPR21889, UK), DVL1 Polyclonal antibody (Proteintech, 27384-1-AP, China), GSK3*β* Polyclonal antibody (Proteintech, 22104-1-AP, China), Anti-beta Catenin antibody(Abcam ab32572, UK), Morris water maze, panoramic biopsy scanner, brain stereotaxic instrument, gel scan imaging, and image analysis system,

### 2.2. Preparation of A*β* Amyloid Protein

The samples were diluted to 1 mg/ml with sterile PBS solution and incubated at 37°C for 1 week to form polymerized A*β* for later use.

### 2.3. Preparation of Lycopene-Loaded Microemulsion and Traditional Lycopene

Polysorbate 80 and Transcutol Hp were prepared at a 2 : 1 ratio, then nitrogen-sealed, vortexed for 5 min, avoiding light, and then mixed at low speeds with a magnetic stirrer for 30 min. Then, we thoroughly incorporated the Smix formation with lycopene (463 *μ*g/ml) into limonene. The mixture was gently shaken using a nitrogen inflator while blending to filter impurities. Next, the Smix was added; then, it was nitrogen-sealed, avoiding light. Afterward, it was placed in a 37°C warm bath and mixed at low speeds with a magnetic stirrer for 30 min. At the same time, syringes for the intravenous drip with a corresponding amount of deionized water (18°*Ω*) were nitrogen-sealed and mixed with a magnetic stirrer for 30 min at 4°C, avoiding light. After 1 day, the transparent crystal LME was available [6]. Traditional lycopene (LOO) was prepared by dissolving the same amount of lycopene in olive oil [6].

### 2.4. Establishment of AD Cognitive Impairment Model in Rats

After the rats were anesthetized by intraperitoneal injection of 2% pentobarbital, the cranial-top hair was shaved to expose the skin, and the rats were fixed on the stereotactic locator. The skin was cut open to expose the skull, and the left lateral ventricle was positioned as the injection target area according to the “stereotactic map of rat brain.” With bregma point as the origin, the skull was drilled with a side opening of 1.3 mm and a back opening of 0.8 mm. A 4 mm microsyringe needle containing A*β* 10 *μ*l (>10 min) was then injected slowly, and the needle core was withdrawn slowly after 5 min of needle retention. Afterward, the opening was sutured, and single cage feeding was performed after surgery until the rat was fully awake.

### 2.5. Animal and Intervention

An animal laboratory provided a total of 24 healthy male Wistar rats (Liaoning Changsheng Biotechnology Co., Ltd.) weighing 150–200 g. The rats were fed adaptively for 1 week. Then, the rats were completely randomly divided into 4 groups: (1) the blank control group (CON group), with no special treatment and nasal feeding with the same amount of normal saline; (2) the AD control group (AD group), injected with A*β* into the lateral ventricle and fed with the same volume of normal saline; (3) the LOO group, injected with A*β* into the lateral ventricle and fed with traditional lycopene; and (4) the LME group, injected with A*β* into the lateral ventricle and fed with LME. The LOO and LME groups were fed with the same amount of LOO and LME (calculated by the content of lycopene, 4 mg/kg/day for each rat). Nasal feeding lasted for 3 weeks in a clean environment, with clean water and a proper diet.

### 2.6. Morris Water Maze Behavior Test

The spatial memory learning ability of rats was measured using the Morris water maze behavioral test. First, the localization cruise experiment was conducted. Here, the rats were immersed into the water from 4 different directions (north, south, east, and west) 4 times a day for 4 days, and the time to find the platform (escape incubation period) and swimming path were recorded within 2 min. If the rat did not find the platform within 2 min, the escape incubation period was recorded as 120 s, and the rats were guided to the platform and placed there for 15 s. The learning ability of the rats could be observed by training the rats to find the platform. On the fifth day of the test, the platform was removed, and two relatively distant entry points were selected for entry into the water. Herein, a space exploration experiment was conducted, which recorded the following variables: time to reach the platform quadrant, times the platform was crossed, and the percentage of time staying in each quadrant.

### 2.7. Sample Sampling

Intraperitoneal injection of 2% pentobarbital was given. Hemostatic forceps were placed, using rat leg pain avoidance reaction as the standard. The chest was cut open, exposing the heart; then, a syringe was used to pierce the rat cardiac apex. Throbbing occurred. The right auricle was cut, and physiological saline was given for heart perfusion until the liver of rats started to bleach. The liquid crystal stop was avoided until after infusion. The rat was beheaded, and the brain was frozen. The hippocampus was separated and frozen using liquid nitrogen, then later cryopreserved for standby at a −80°C refrigerator. The heart was given 4% paraformaldehyde perfusion; then, the hippocampus was harvested after sliced paraffin embedding (*d* = 3 *μ*m) for immunofluorescence staining.

### 2.8. qRT-PCR Was Used to Detect Genes Related to Neurogenesis

The total RNA was isolated from the hippocampal tissues using TRIzol (Thermo, 15596026, USA) according to the manufacturer's instructions. A reverse transcription kit (Takara, RR047A, Japan) was used to reverse-transcribe RNA to cDNA. Next, qRT-PCR was performed using cDNA as a template on a Light Cycler system with FastStart DNA Master SYBR Green *Ι* (Roche, 03003230001, Germany). The primers used were as follows: 36B4, 5′-CACTGGTCTAGGACCCGAGAAG-3′ and 5′-GGTGCCTCTGGAGATTTTCG-3′; Pax6, 5ax6, TGCCTCAGCATGCAGAACAGTCAC-3′ and 5nd6, TGCCTCAGCATGCAGAACAGTCAC-3′; Reelin, 5′-GCGTGCTGCTGGACTACTCT-3′ and 5′-GAAATCCATCTCATGAAGCAAA-3′; and Nestin, 5estin, ATCCATCTCATGAAGCAAA- and 5dtin, ATCCATCTCATGAAGCAAA-3.

The difference between the Ct values (*Δ*Ct) of the gene of interest and the housekeeping gene was calculated for each experimental sample. Then, the difference in the *Δ*Ct values between the experimental and control sample *ΔΔ*Ct was calculated. The fold-change in the expression of the gene of interest between the two samples was equal to 2^-*ΔΔ*Ct^.

### 2.9. Western Blotting to Detect the Expression of Wnt3a, *β*-Catenin, GSK3*β*, and p-GSK3*β* in the Hippocampus

The proteins were extracted according to the BCA extraction kit instructions, and the samples were loaded for denaturing discontinuous polyacrylamide gel electrophoresis (SDS-PAGE) then transferred to the PVDF membrane. The PVDF membrane was soaked in a blocking solution of 5% skimmed milk powder for washing. After washing, the PVDF membrane reacted with the corresponding primary antibody and then incubated overnight in a 4°C refrigerator and shaker. After rinsing the next day, the corresponding secondary antibody was added and incubated at room temperature for 1 hour. The newly configured chromographic solution was then for photographic preservation.

### 2.10. Tissue Fluorescence Staining to Detect Neurogenesis in the Hippocampus

Sections were routinely dewaxed, microwave antigens were repaired, and the 3% bovine serum was sealed for 30 minutes. Next, the corresponding primary antibodies of BrdU, Neun, Dcx, and Iba1 were added, and the sections were incubated overnight in a wet box at 4°C. The second antibody was added after PBS rinsing the next day, and the reaction was conducted at room temperature for 1 hour. The second antibody was decanted then rinsed with PBS. The nucleus was stained with DAPI, the slices were sealed, and then, the fluorescence section scanner was used to take photos. The corresponding color of positive cells was shown, and the colabeled cells were considered double-positive cells. The negative control was 3% BSA instead of the primary antibody.

### 2.11. Statistical Analysis

Data were expressed as mean ± SEM and standard deviation. One-way ANOVA was performed using the SPSS11.0 statistical software, followed by the Tukey-Kramer post hoc multiple comparison test. Test level *α* = 0.5 and *p* < 0.05 indicated statistically significant difference.

## 3. Results

### 3.1. Behavioral Test of Rats (Figure 1)

**Figure 1:**
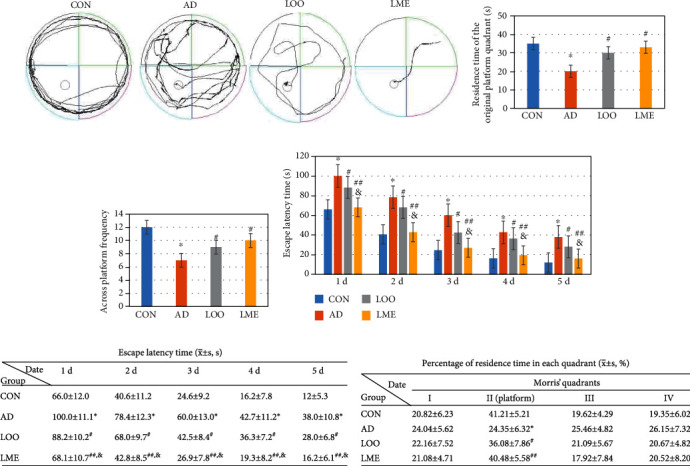
Effect of LME on spatial memory and learning ability in rats. (a) Trajectory map of rats. The trajectory of untrained rats was mainly marginal. The trained rats' path to find the platform gradually shortened. (b) Residence time of the original platform quadrant of the CON-, AD-, LOO-, and LME-treated rats. The AD group had a shorter time than the control group. This indicates that the AD model is successful. The LOO-treated rats had a longer time than the AD group, while compared with the AD group, the LME group had much longer time. (c) Across platform frequency of rats. The times of crossing the platform in LME group was close to that in the control group. This indicates that LME could effectively improve the spatial memory and learning ability in AD rats. (d, e) Escape latency time of four group rats. The AD group had longer than the CON group. The LOO and LME groups are significantly shorter than the AD group. Compared with the AD group, the LME group could make much shorter escape latency time. (f) Percentage of residence time of the platform quadrant. Values are expressed as mean ± SEM (*n* = 6 rats/group). ^∗^*p* < 0.05 versus the control group. ^#^*p* < 0.05 versus the AD group. ^##^*p* < 0.01 versus the AD group. ^&^*p* < 0.05 versus the LOO group.

In the Morris water maze directional cruise experiment, the average escape latency time of rats in each group gradually shortened (Figures 1(d) and 1(e)), indicating that the ability to find the platform improved after learning and training. In 2 d, when comparing the AD and CON groups, the escape latency was significantly prolonged (Figures 1(d) and 1(e)). The trajectory of rats was mostly marginal and random (Figure 1(a)), indicating that the modeling was successful. After removing the platform, the AD group had significantly shortened platform quadrant time than other groups (Figures 1(b) and 1(f)), suggesting the effects of learning and memory impairment. Compared with the LOO group, the escape latency of the LME group was significantly shorter (Figures 1(d) and 1(e)), but both the LOO and LME groups were significantly shorter than the CON group. There was also no significant difference between the LME and CON groups. These results indicate that LOO and LME can improve AD rats' spatial learning and memory ability, with a more significant improvement with LME (Figures 1(d) and 1(e)).

### 3.2. Detection of Neural Proliferation-Related Genes on qRT-PCR (Figure 2)

The CON and AD groups were not significantly different in detecting Pax6, Nestin, and Reelin. On the other hand, LOO increased the expression of these genes. Furthermore, the LME group had significantly increased expression of Pax6 and Nestin compared to the LOO group. Thus, LOO and LME had positive effects on Reelin, Nestin, and Pax6 expressions in the rat hippocampus, with LME having a more positive effect on the expression of these genes. Thus, LME likely regulates neurogenesis by increasing the expression of Pax6, Nestin, and Reelin genes.

### 3.3. Nerve Proliferation in the Hippocampus (Figure 3)

**Figure 3:**
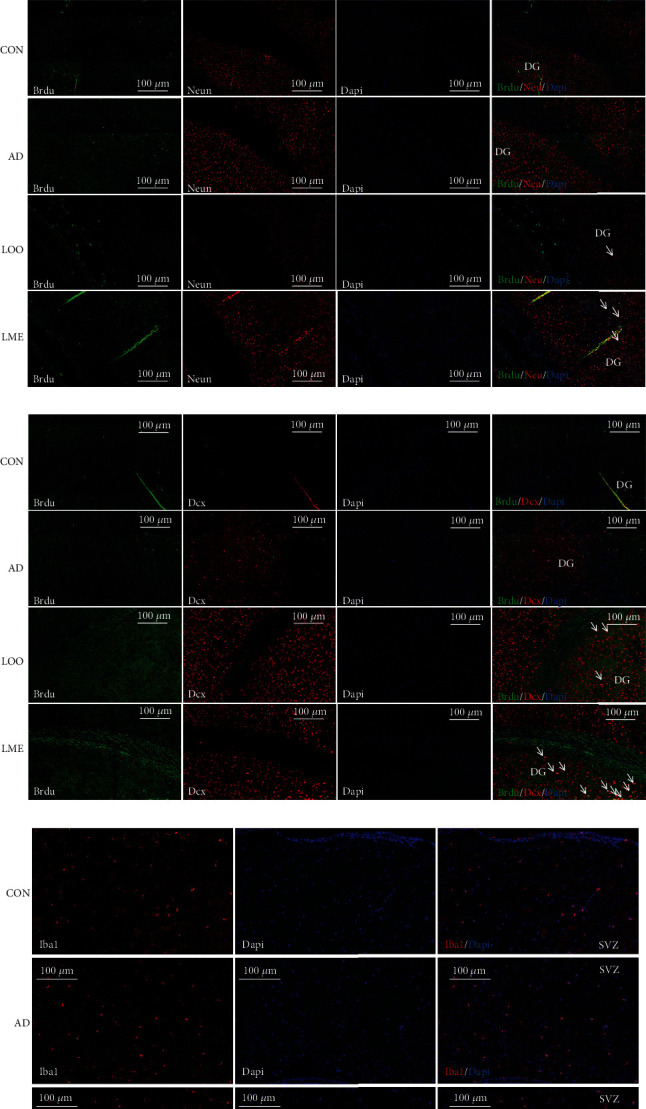
LOO and LME increase neuronal differentiation and adult neurogenesis in the hippocampus and SVZ of rats, especially LME. Dcx (red: marker for adult neurogenesis); BrdU (green; proliferating marker); NeuN (red; mature neuronal marker); Iba1(red; microglia marker).(a) Photomicrographs showing immunostaining of BrdU^+^ cells in the region of SVZ. There were almost no BrdU^+^ cells in the CON group. Scattered BrdU+ cells were found in the AD group, while the LOO group could see many BrdU^+^ cells. A large number of BrdU^+^ cells were observed in the LME group. LME significantly promoted neurogenesis in AD rats. (b) Photomicrographs showing immunostaining of Dcx^+^ cells in the dentate gyrus region of the hippocampus. The number of Dcx^+^ cells in the LOO group was higher than that in the CON and AD groups and in LME group was higher than that in the LOO group. (c, f) Quantification analysis suggested a significantly increased number of mature neurons with BrdU in the SVZ and dentate gyrus region of the hippocampus of LOO- and LME-treated rats, especially LME-treated rats. (d) Quantification analysis of Dcx^+^ cells in the dentate gyrus region of the hippocampus. (h) Double immunofluorescence analysis of newly born neurons colabeled with Dcx and BrdU in the dentate gyrus region of the hippocampus of CON-, AD-, LOO-, and LME-treated rats. The LME group can see a lot of colabeled with Dcx and BrdU cells. Arrows indicate BrdU+ nuclei colabeled with Dcx. (i) The LOO group can see many BrdU^+^ cells and colabeled with BrdU and Neun in the dentate gyrus region of the hippocampus; the LME group can see more BrdU^+^ and BrdU^+^/Neun^+^ cells. LME promotes the differentiation of newborn neurons into mature neuronal. (e, g) Quantitative analysis in the hippocampal sections showed a significantly increased number of immature neurons colabeled with BrdU^+^/Dcx^+^ and BrdU^+^/Neun^+^, suggesting increased neuronal differentiation in LME-treated rats. (j, k, o) Photomicrographs showing immunostaining of Iba1^+^ and Iba1^+^/BruU^+^ colabeled cells in the region of SVZ. (l–o) Immunostaining of Iba1^+^ and Iba1^+^/BrdU^+^ colabeled cells in the dentate gyrus region of the hippocampus. (m, n) A small number of Iba1^+^ and Iba1^+^/BruU^+^ cells in the CON group. Compared with the CON group, the number of Iba1^+^ and Iba1^+^/BruU^+^ colabeled cells were significantly increased. Compared with the AD group, the number of positive cells of the LOO group was decreased. Compared with the LOO group, the number of positive cells of the LME group was even lower. Values are expressed as mean ± SEM (*n* = 6 rats/group). ^∗^*p* < 0.05 versus the AD group. ^∗∗^*p* < 0.01 versus the AD group. Scale bar = 100 *μ*m. ^#^*p* < 0.05 versus the CON group. ^&^*p* < 0.05 versus the LOO group.

In the SVZ region of the CON and AD groups, the number of BrdU^+^ cells was very small. Similarly, a small amount of BrdU^+^ cells was in the LOO group, but this number significantly increased in the LME group (Figures 3(a) and 3(c)). In the DG region, the Dcx^+^ cells were almost invisible in the CON group, while a small amount was found in the AD group (Figures 3(b) and 3(d)). Compared to the CON and AD groups, many BrdU^+^, Dcx^+^, and BrdU+/Dcx^+^ colabeled cells were observed in the LOO and LME groups (Figures 3(h) and 3(e)), with the latter having a significant increase than the former (Figures 3(h) and 3(g)). Compared to the CON and AD groups, a small amount of BrdU^+^/Neun^+^ colabeled cells was observed in the LOO group, with a significantly higher number in the LME group compared to the LOO group (Figures 3(i) and 3(e)). Thus, lycopene could promote neurogenesis to a certain extent, while the effect of LME on neurogenesis was more significant.

A small number of Iba1^+^ and Iba1^+^/BrdU^+^ colabeled cells were observed in the CON group, while many were observed in the AD group. However, compared with the AD group, these cells were significantly reduced in the LOO and LME groups, with a more obvious decrease in the LME group (Figures 3(j)–3(m)). Thus, LME can reduce the neuroinflammatory response.

### 3.4. Effect of Lycopene Nanoemulsion on the Protein Expression of Wnt Pathway in the Hippocampus Seen on Western Blot (Figure 4)

Compared to the CON group, the protein expressions of Wnt3a, *β*-catenin, and Dvl in the AD group increased, while the protein expressions of P-*β*-catenin and GSK3*β* decreased. Compared to the AD control group, the LOO group had increased protein expressions of Wnt3a, *β*-catenin, and DVL and decreased protein expressions of p-*β*-catenin and GSK3*β*. In the LME group, the trend was more significant, and the differences were statistically significant. These results indicate that the proteins associated with the Wnt pathway changed in each group. Compared with the CON group, the other 3 groups showed elevations in active proteins (Wnt3a, *β*-catenin, and Dvl) and reductions in inhibitory proteins (P-*β*-catenin and GSK3*β*). Thus, it can be inferred that the Wnt pathway may be involved in the neurogenesis of A*β*-induced AD rats.

## 4. Discussion

The Morris water maze is an experimental method designed by the British psychologist Morris in 1981. It is used to study brain learning and memory mechanisms, and its application in AD research is very common [3]. The more classic Morris water maze test procedure mainly includes a positioning sea trial and space exploration test in two parts. In this study, the LOO group had shortened escape latency time and more frequently crossed the platform than the AD group. Furthermore, these variables were significantly different when comparing the LOO group with the LME group. Thus, LME could effectively improve the spatial learning and memory impairment of A*β*-induced AD rats.

Reelin is a secreted glycoprotein and extracellular matrix protein involved in brain development, synaptic plasticity, learning and memory, NSC proliferation, neurosphericity, and neuroblast migration [8, 9]. The absence of Reelin has a negative effect on the proliferation of NSC [8]. Reelin-mediated reduction in signal transduction is associated with the pathogenesis of various neurodegenerative diseases, including AD and other age-related diseases [9, 10]. Nestin-intermediate filament protein is necessary for NSC proliferation and self-renewal [11]. Pax6 is another gene that is highly expressed in neural stem cells and plays an important role in neurogenesis [12, 13]. The high expression of Pax6 in the hippocampus is necessary for the generation and maintenance of neurogenesis [14, 15]. In our study, LOO and LME had positive effects on Reelin, Nestin, and Pax6 expressions in the hippocampus of rats, with the LME group demonstrating a more distinct expression level. Thus, LME likely promotes neurogenesis by increasing the expression of this gene.

BrdU, also known as 5′-′bromo-2′-deoxyuridine, is a thymidine analog, which competitively binds to DNA with endogenous thymidines. Studies have shown that once bound to DNA, BrdU persists and is gradually passed on to the next generation as cells divide. BrdU-labeled neurons can be detected days, weeks, months, or even years after the fixation of brain tissue with specific antibodies [16]. Typically, mice injected intraperitoneally with BrdU can pass the blood-brain barrier, which is the gold standard for research related to nerve cell regeneration. Simply put, BrdU markers can detect new cells in neurogenesis [17–19]. Dcx, also known as doublecortin, is widely expressed in migratory neurons and can also be observed early in neuronal development. It is a microtubule-related protein whose antibody can specifically mark the cytoplasm and processes of neural precursor cells. Dcx was detected on the 3rd day after injecting a virus with transcription factor Neuord1 in Parkinson's model mice [20] and in a MCAO model, Dcx can also be detected on the 7th day [20]. Studies have confirmed that Dcx is a reliable and specific marker reflecting the level of adult neurogenesis and its regulation. It has become a widely used marker in the analysis of adult neurogenesis [21, 22]. In our study, a large number of new BrdU^+^, Dcx^+^, and BrdU^+^/Dcx^+^ cells were observed in the LME group, suggesting that LME significantly promotes neurogenesis.

Neuroinflammation is one of the major pathological changes in patients with AD, which can accelerate the deterioration of the disease. A*β* deposition can activate astrocytes and microglia, leading to the release of various proinflammatory factors and further promoting A*β* accumulation, neuronal degeneration, and dysfunction [23, 24]. Astrocytes and microglia were abundant in the brains of autopsied AD patients [25], and the same site was observed in A*β*1-42 injected rats [26]. Iba1, also known as Ionized calcium-binding adapter molecule 1, is a specific protein antibody of the microglia and macrophage. Iba1 is expressed in and increased during the activation of these cells. LME significantly reduced the number of Iba1^+^ and Iba1^+^/BrdU^+^ cells in the DG and SVZ regions, suggesting that LME could significantly reduce the neuroinflammatory response.

Wnt is a type of protein that widely exists in the secretion of organism protein growth factor (either through the autocrine or paracrine route) and in the related receptors on the surface of the cell membrane. It activates the cell signaling pathways and regulates the corresponding target gene expression. It is important in the embryonic development of various kinds of cell proliferation, differentiation, migration, and apoptosis [27, 28]. The Wnt signaling pathway is mainly composed of the Wnt protein family of extracellular factors: frizzled (FZD) protein, Dvl protein, adenomatous polyposis coli gene products, GSK-3*β*, *β*-catenin, Axin, T-cytokine/lymphoid enhancement factor (TCF/LEF), and other factors. The Wnt signaling pathway plays an important role in the development of the nervous system, which is closely related to neural tube development and ganglion formation. It can induce axon growth, dendrite formation, and protrusion and participate in the regulation of forebrain formation during the development of the central nervous system. The Wnt signaling pathways are divided into the classical Wnt/*β*-catenin signaling pathway and nonclassical signaling pathway according to different modes of action [29, 30]. In the classic Wnt/*β*-catenin signaling pathway, extracellular proteoglycan promotes Wnt signaling in the cytoplasm. The Wnt/*β*-catenin signaling pathway then activates the cytoplasmic Dvl protein through the FZD family. Studies have shown that the loss of Wnt3a leads to the loss of the entire hippocampus [31]. This defect may be caused by the changes of the Wnt/*β*-catenin pathway that interfere with NSC proliferation. This inhibits the activity of GSK-3*β*, which in turn inhibits the degradation of *β*-catenin, which continuously accumulates in the cytoplasm. Finally, the Wnt signal is transmitted into the cell to perform its corresponding function [30]. The Western blot analysis in our study proved that LME might regulate neurogenesis by activating the Wnt/*β*-catenin pathway to compensate for nerve loss caused by AD.

Studies have shown that lycopene-loaded microemulsion(LME) has excellent physical and chemical properties, higher oral bioavailability, and superior brain-targeting ability [8, 32]. These findings provide the basis for the application of oral brain-targeted drug delivery strategies. We further studied the promotion effect of lycopene-loaded microemulsion on cognition and neurogenesis in A*β*-induced Alzheimer's rats and the possible signaling pathway, This may providing a new idea for the treatment and research of Alzheimer's disease.

## Figures and Tables

**Figure 2:**
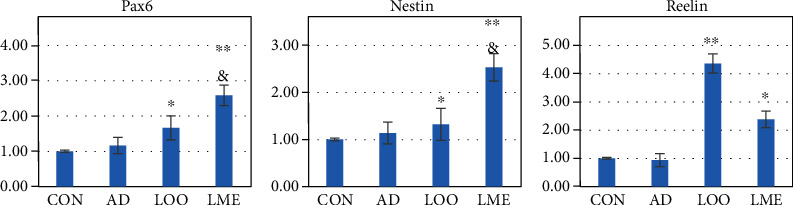
(a–c) Quantitative real-time PCR analysis was performed for relative mRNA expression of nestin (marker of NSC), Reelin (an extracellular matrix protein required for proper migration and differentiation of NSC), and Pax6 in the hippocampus and normalized to *β*-actin. Values are expressed as mean ± SEM (*n* = 6 rats/group). Compared with the control group, the expression of nestin, Reelin, and Pax6 of the AD has no obvious change (*p*>0.05). Compared with the AD group, the expression of Pax6 and nestin in the LOO and LME groups is significantly increased. Compared with the LOO group, the expression of Pax6 and nestin is significantly increased. ^∗^*p* < 0.05 versus the AD group. ^∗∗^*p* < 0.05 versus the AD group. ^&^*p* < 0.05 versus the LOO group.

**Figure 4:**
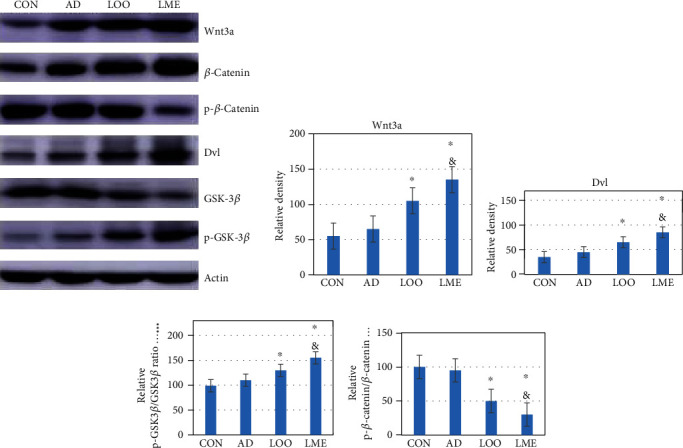
LME increases neurogenesis in the hippocampus through activation of the Wnt/*β*-catenin pathway. (a) Western blot analysis of Wnt3a, Disheveled, GSK-3*β*, p-GSK-3*β*, *β*-catenin, and p-*β*-catenin protein levels in the hippocampus. Values were normalized to *β*-actin. (b, c) Quantification of relative protein density after normalization with *β*-actin. (d, e) Ratio of p-GSK-3*β*/GSK-3*β* was significantly increased, and p-*β*-catenin/*β*-catenin significantly decreased in the LOO- and LME-treated rats. Representative blots showing two samples from each group. Mean ± SEM (*n* = 6 rats/group). ^∗^*p* < 0.05 versus the AD group. ^&^*p* < 0.05 versus the LOO group.

## Data Availability

The data that support the findings of this study are available from the corresponding author upon reasonable request.
